# Supplementary material for: Participation of young women in sexual and reproductive health decision-making in Malawi: Local realities versus global rhetoric
Source: PLOS Glob Public Health. 2022 Nov 23;2(11):e0001297. doi: 10.1371/journal.pgph.0001297 (PMC10022123; doi:10.1371/journal.pgph.0001297)
Supplement: S1 File — (DOCX) [file pgph.0001297.s001.docx]

Inclusivity in global research

PLOS’ policy on inclusivity in global research aims to improve transparency in the reporting of research performed outside of researchers’ own country or community and ensures that PLOS publications reporting global research adhere to high standards for research ethics and authorship. Authors of relevant research articles may be asked to complete the questionnaire below, which outlines ethical, cultural, and scientific considerations specific to inclusivity in global research. This questionnaire may be requested when researchers have travelled to a different country to conduct research, if research uses samples collected in another country, research with Indigenous populations or their lands, or if research is on cultural artefacts. Researchers travelling to another country solely to use laboratory equipment will not normally be required to complete the questionnaire. However, the questionnaire can be requested at the journal’s discretion for any submission – if you have been requested to complete this questionnaire by the PLOS journal you submitted to, please do so.

Please complete the questionnaire below and include this as a Supporting Information file with your manuscript. Note that if your paper is accepted for publication, this checklist will be published with your article in the supporting information files. Please ensure that you reference the checklist in the main body of your manuscript. We suggest adding a subsection ‘Inclusivity in global research’ to your Methods section and adding the following sentence: “Additional information regarding the ethical, cultural, and scientific considerations specific to inclusivity in global research is included in the Supporting Information (SX Checklist)”

The questions have been designed to be applicable to a wide range of study types, and there are subsections for both human subjects research and non-human subjects research. If any of the questions are not relevant to your research please mark them as “N/A” as appropriate.

**Ethical considerations, permits and authorship**

*This section is applicable to all research types.*

Provide details as to who granted permissions and/or consent for the study to take place in the Methods section of your manuscript. This should include the names of **all** ethics boards, governmental organizations, community leaders or other bodies that provided approval for the study. If individuals provided approval refer to these people by their role or title but do not list their name(s).

Reported on page number: Details of ethics boards (University of Toronto and the National Health Sciences Research Committee) that approved this study are outlined on page 13 in the materials and methods section.

If there were any deviations from the study protocol after approval was obtained please provide details of these changes in the Methods section of your manuscript.
Did this study involve local collaborators that are residents of the country where the research was conducted or members of the community studied? If you do not have any authors from said communities, please provide an explanation for this below.

A minor protocol deviation occurred as we experienced greater than anticipated interest for recruitment of youth to participate in focus group discussions. As a result, we increased the number of FGD participants from 4-5 individuals to 5-10 for each FGD. This involved both a deviation, and amendment submitted and approved by the University of Toronto in November 2017.

Reported on page number: 13

**Yes, this study involved local collaborators. Specifically, Stewart Paul, Monica Kalolo and Lumbani Banda were Malawian research team members.**

Everyone listed as an author should meet PLOS’ criteria for authorship and all individuals who meet these criteria should be included in the author byline, rather than the acknowledgements. Authorship criteria is based on the International Committee of Medical Journal Editors (ICMJE) Uniform Requirements for Manuscripts Submitted to Biomedical Journals - for further information please see here: <https://journals.plos.org/plosone/s/authorship>.

**We confirm that all authors listed on this manuscript meet the criteria for authorship as outlined in this resource.**

**Human subjects research (e.g. health research, medical research, cross-cultural psychology)**

Did you obtain written informed consent from a representative of the local community or region before the research took place? How did you establish who speaks for the community? Details of written informed consent obtained from study participants should be reported separately in the Methods section of your manuscript.

Written informed consent was obtained from the three focal districts (Dowa, Nkhata Bay and Zomba) to conduct research in these areas. This is outlined in the materials and methods section on page 13.

How did members of the local community provide input on the aims of the research investigation, its methodology, and its anticipated outcome(s)?

When engaging with the local community, how did you ensure that the informed consent documents and other materials could be understood by local stakeholders?

All informed consent documents and research materials were available in both English and Chichewa. All participants selected their preferred language for research materials, and translation and interpretation was offered.

This research is informed by the first author’s (JW) previous global health work experience. She also conducted a formative research trip in Malawi (in March-April 2017) prior to writing the research proposal, which offered an opportunity to gain feedback on the research topic, aims and proposed methodology from our host institution (the Parent and Child Health Initiative – PACHI), and to hold informal meetings with stakeholders working in youth SRH (e.g., local and international NGO/CSO representatives). We also engaged a youthful Malawian research assistant (SP) and six youth researchers to share power through co-constructing knowledge within a space of mutual influence that extends beyond our respective cultures, age and gender.

Will the findings of the research be made available in an understandable format to stakeholders in the community where the study was conducted (e.g. via a presentation, summary report, copies of publications, etc.)? Please provide details of how this will be achieved.

A policy brief written in lay language and available in both English and Chichewa was developed and distributed to all research participants in both print and electronic forms to share preliminary research findings in May 2018. The first publication from this study (Wigle, et al., 2020 “Youth participation in sexual and reproductive health: policy, practice, and progress in Malawi”) was also shared to all key informants and many youth participants via email and Whatsapp. We also plan to share future publications from this research and are exploring other alternatives to ensure our findings shared with diverse stakeholders (e.g., online presentations/webinars, collaborating with our host institution PACHI and sharing with youth researchers and youth). For example, SP currently works at a youth-led NGO based in Malawi, and will help to ensure our findings are accessible and shared with young people.

**Non-human subjects research using specimens/ animals collected as part of the study, or those housed in archival collections. Examples include archaeology, paleontology, botany and zoology.**

Did the permission you obtained from a local authority to perform the study include an agreement on access to outputs and benefit sharing? This may include procedures to enable fair distribution of the benefits and resources arising from the research performed. Please include any details of Prior Informed Consent and Benefit Sharing Agreements obtained. These may be required by field-specific regulations, for example the Convention on Biological Diversity (CBD) and the associated Nagoya Protocol.

N/A

If the material used in your study was imported, please A) provide the year it was imported and B) indicate whether permits were obtained to import/export the materials used, C) provide details of any permits obtained. If this information is not available, please indicate this.

N/A

If you used archival specimens, please state how the material used in your study was acquired by the institute it is held in and provide details of any permits obtained for the original excavations/ sample collection. If this information is not available, please indicate this.

N/A

How was the potential cultural significance of the materials collected in your study to local communities considered in your research design? Were Indigenous peoples and/or local researchers and institutions involved with archaeological excavations / collection of specimens? If so, please provide a description of their involvement.

N/A

If your manuscript includes photographs of human remains please indicate whether authors obtained permission from descendants or affiliated cultural communities to do so.

N/A
