# Supplementary material for: Participation of young women in sexual and reproductive health decision-making in Malawi: Local realities versus global rhetoric
Source: PLOS Glob Public Health. 2022 Nov 23;2(11):e0001297. doi: 10.1371/journal.pgph.0001297 (PMC10022123; doi:10.1371/journal.pgph.0001297)
Supplement: Attachment — Submitted filename: PLoSGPH Response to Reviewers 08-09-2022.docx [file pgph.0001297.s002.docx]

**Response to Reviewers**

Please find below a detailed summary of reviewer comments, and responses/changes made to address feedback.

**Table: Summary of Journal Requirements**

| **Journal Requirements** | **Responses** |
| --- | --- |
| **Introduction** | |
| 1. Please include a complete copy of PLOS’ questionnaire on inclusivity in global research in your revised manuscript. Our policy for research in this area aims to improve transparency in the reporting of research performed outside of researchers’ own country or community. The policy applies to researchers who have travelled to a different country to conduct research, research with Indigenous populations or their lands, and research on cultural artefacts. The questionnaire can also be requested at the journal’s discretion for any other submissions, even if these conditions are not met.  Please find more information on the policy and a link to download a blank copy of the questionnaire here: https://journals.plos.org/globalpublichealth/s/best-practices-in-research-reporting. Please upload a completed version of your questionnaire as Supporting Information when you resubmit your manuscript. | - Thank you for sharing this resource. A copy of our completed questionnaire was uploaded as supporting information in our resubmission. |
| 2. Please amend your detailed Financial Disclosure statement. This is published with the article. It must therefore be completed in full sentences and contain the exact wording you wish to be published.  2a. State the initials, alongside each funding source, of each author to receive each grant. | Updated financial disclosure statement:  This research was supported by the International Development Research Centre under Grant #108279-010 (JW) and the Social Sciences and Humanities Research Council of Canada Grant #752-2017-1555 (JW). The funders had no role in study design, data collection and analysis, decision to publish, or preparation of the manuscript. |
| 3. Please provide separate figure files in .tif or .eps format and removed from the manuscript file.  For more information about figure files please see our guidelines:  https://journals.plos.org/globalpublichealth/s/figures  <https://journals.plos.org/globalpublichealth/s/figures#loc-file-requirements> | Figure files in .tif format are now provided. |
| 4. Figs 1-3: Please confirm (a) that you are the photographer; or (b) provide written permission from the photographer to publish the photo(s) under our CC-BY 4.0 license | We confirm that Jannah Wigle (corresponding author) is the photographer of photo in Figure 1.  Figures 2 and 3 represent drawings produced by youth participants and photographed by Jannah Wigle. |
| 5. We have noticed that you have uploaded Supporting Information files, but you have not included a list of legends. Please add a full list of legends for your Supporting Information files after the references list. | We did not upload supporting information files, no change was made. |
| 6. In the online submission form you indicate that your data is not available for proprietary reasons and have provided a contact point for accessing this data. Please note that your current contact point is a co-author on this manuscript. According to our Data Policy, the contact point must not be an author on the manuscript and must be a third party. Please revise your data statement to a non-author institutional point of contact, such as a data access or ethics committee, and send this to us via return email. Please also include contact information for the third party organization, and please include the full citation of where the data can be found. | We have revised our data availability statement and would like it to now state:  The data that support the findings of this study are not publicly available due to the ethics consent form indicating that participant data would not be shared outside of the research team. This study is based on extensive identifiable data from observations, interviews and focus group discussions. These data may contain information that could compromise the trust, confidentiality, and privacy of research participants, and thus will not be made publicly available. This research study was approved by the Research Ethics Board at the University of Toronto. If you have any questions or concerns or requests for data access, please contact the Research Oversight and Compliance Office – Human Research Ethics Program at [ethics.review@utoronto.ca](mailto:ethics.review@utoronto.ca) or by phone at +1 (416) 946-3273. |

**Table: Summary of Peer Reviewer Feedback & Responses**

| **Reviewer #1 Comment** | **Responses** |
| --- | --- |
| **Introduction** | |
| 1. The title of this is about SRH decision-making, but sometimes it is not always clear how you are defining participation and decision-making (which can have an enormous array of meanings in SRHR research). 2. Line 37 – make explicit that this is not about (as far as I understand it) participation in daily SRH decisions? But is about SRH at the political level. | - Thank you for this comment! To provide further context for our understanding of key terms, we have added our definition and understanding of: “youth participation”, “policymaking” and “decision-making” to Table 1. Importantly, this helps to clarify our understanding and application of participation vs decision-making. - This was clarified and now states: “…to explore how young women’s complex realities of ***participating in SRH policymaking*** in Malawi are shaped by underlying structural and societal forces.” |
| 3. It could be worth acknowledging that the category of youth, as defined in Malawian policy, also allows for flexibility in age markers (<https://www.unicef.org/esa/media/9031/file/UNICEF-Malawi-2020-2021-Youth-Budget-Brief.pdf>)   In a later point (line 493) you talk about when a young woman is considered a youth, and how this intersects with her sexual and reproductive lifecourse. Grappling with this could be brought out further, particularly the incongruity of what the government determine to be a youth compared to the contextual realities (in which markers other than age are involved).  Recommend reading African Sexualities: A Reader (ed. Sylvia Tamale) – has interesting and important work on gender and sexuality across Africa | - We have added the language used in the *National Youth Policy* (2013) to highlight that the definition of “youth” is “quite flexible”. - Thank you for this suggestion. We have added the following text to reflect this feedback on page 28: ***This highlights the disconnect between the definition of “youth” in the National Youth Policy, as compared to young women’s lived, contextual realities of participation.*** - Thank you for sharing this resource. We have read this reference and added a citation for Sylvia Tamale’s work to support our claims relating to the preservation of patriarchal control over young women’s bodies in the discussion section |
| 4. Line 210: six youth researchers engaged – how? What was this process? Could there be any reflection on the potential age differences between youth researchers and participants? | - To address this feedback we added details on the recruitment of youth researchers: “Youth researchers were ***recruited via the host institution’s website, social media and local networks, and the district youth offices in each region****.* ***Candidates were interviews and selected, and*** *provided basic training in qualitative methods…”* - Youth researchers *and* participants were all aged 16-24 years. There was limited age differences between youth researchers and participants. Some youth researchers/participants belonged to the same youth clubs, university programs, etc. as participants and therefore recruited their peers to participate in the FGDs/interviews. We added that youth researchers supported disseminating the recruitment poster ***with their peers and network****s* to highlight the similarity in ages. Therefore, we do not provide a reflection on the potential age differences between youth researchers and participants. |
| 5. 257-58: What is meant by generating data “from youth and key informants’’ perspectives”? | - This was removed to improve clarity. |
| **Methods** | |
| 6. Some of the methods that you use feel underdeveloped in the paper.  The document analysis of policies in particular feels underused, and it reduces the impact of the novelty of your work. Taking your theoretical framework and use of an intersectional lens in your analysis, what do the documents tell us specifically about e.g., how ‘youths’ are defined, how do policies talk about women, who is included / excluded / not mentioned? Extending your theoretical approach in your document analysis would bolster the discussion, which at times does not feel as novel as it could. | - Thank you for this comment. We previously published the results of our document analysis (see Wigle, et al. 2020), which outlined how youth are defined in various youth/SRH/health policies and whether the policies indicated whether youth were engaged and/or consulted in the policymaking process. However, we agree that more explicitly discussing the findings from the document analysis in this paper would be beneficial for readers. To this end we have added several sentences: - Page 20 (results): ***Furthermore, few national youth, SRH or health policies offer data on youth participation or SRH disaggregated by sex, nor acknowledged the systemic exclusion of young women in decision-making spaces.*** - Page 29 (results): ***This highlights the disconnect between the definition of “youth” in the National Youth Policy, as compared to young women’s lived, contextual realities of participation.*** - Page 34 (discussion): ***Failure to prioritize young women’s participation within key national policies, such as the National Youth Policy, contributes to fewer opportunities for young women to be engaged in SRH decision-making as youth and perpetuates inequities moving forward*** *(5).* |
| 7. The data from focus groups is often presented similarly to data gathered from in-depth interviews. This doesn’t maximise the uniqueness of focus group discussions at capturing how concepts and topics are discussed between participants, and the insights we can gain from these interactions. | - Our analysis and interpretation is informed simultaneously by multiple research methods employed in this study. - We have chosen not to present findings by individual research method (e.g., FGDs vs interviews) as there were many overlapping concepts/themes. To clarify our decision to discuss and present our findings across methods similarly and simultaneously we have added the following sentence to our analysis section (Page 18): ***Findings from FGDs and interviews are presented together due to the overlapping discussions and themes generated across these methods.*** |
| **Results** | |
| 8. Gender Equality Rhetoric: could this section be linked more specifically to SRH? There is the quote about chiefs saying that girls cannot be leaders – what is this in reference to? Does participation in youth parliament, for example, matter when it comes to SRH? It definitely has an important implication for general political participation, but could be useful to be clear how this relates to your RQs. | - In this instance, we are building our case that gender equality, SRH and youth participation are intimately linked. We emphasize this point in our discussion and conclusion, as young women’s participation in SRH policymaking is not possible without their engagement in broader policy structures. - To more clearly make this connection a sentence was added after the identified quote (citing Chiefs) (Page 21): ***Young women’s participation in SRH decision-making processes is intimately related to and dependent on gender equity and fulfilling their SRH rights.*** |
| 9. Under Donor Influence on Youth and SRH Policy: Why does it matter that there are anti-abortion groups in relation to the Termination of Pregnancy Bill? How does this relate to youth participation in SRH decision-making? | - The sentence describing the role of anti-abortion groups was removed, as it was largely to provide context about local beliefs related to the legalization of abortion. However, we agree that this is not explicitly related to youth participation and removed to improve clarity of this section. |
| 10. 530-532: “Many female youth participants overcame barriers to participate in SRH decision-making, such as adolescent pregnancy, gender-based violence and discrimination from their peers, family and communities.” - What are these barriers? Adolescent pregnancy is represented as a barrier, what if an adolescent is pregnant people they had full SRH decision-making? There’s a value judgement here that needs explaining. | - This comment from the reviewer is a bit difficult to follow. However, it was not our intention to place a value judgment. Several youth emphasized that marriage and/or pregnancy undermined young women’s ability to participate as they are no longer considered “youth” and their continued engagement in youth/SRH spaces was inappropriate and unlikely. - To address this comment, we replaced “barriers” with ***“diverse personal circumstances”***. |
| 11. Quote 548-550 – “the world is promoting women” – ties to earlier points about the global discourse? Does this show the utilisation by young people of global discourses to create power within their community spaces? | - Thank you for this really insightful feedback. We have integrated this comment into the text describing this quote. The following text was added: ***“One female youth participation illustrated the ways that young people have also capitalized on global discourses and momentum around gender equality to create power within SRH decision-making spaces…”*** |
| **Discussion** |  |
| 12. 608-609: “We show that donors’ political and ideological priorities profoundly influence the entire SRH policymaking process” – repeated on 613-614 | - Thank you for catching this duplication. This was removed. |
| 13. 646: “Efforts to address high rates of HIV/AIDS and maternal mortality in Malawi…were associated with an increased public acceptance of SRH issues by study participants” – I don’t think this evidence has been shown? Perhaps speaks to the point below about your methods. | - We have added a sentence (including a quote from a community leader) to support this claim in our results section and more clearly link to our discussion section. The following was added on page 28: ***Several key informants indicated that “in the wake of the HIV pandemic” (Community Leader) HIV/AIDS and SRH remain critical public health priorities, which has helped to decrease the shame or discomfort associated with discussing these issues.*** |
| 14. 748 = “drastic” increases in adolescent pregnancy is potentially stigmatising language. Would be better to show evidence that these pregnancies were not wanted or a choice among adolescents | - The word “drastic” was removed to avoid any stigmatizing language. - We have added further evidence on pages 42-43 to provide support for our statements regarding the recent increases (of 11%) in adolescent pregnancy during the COVID-19 pandemic a rapid assessment report by the Malawi government. We have highlighted the key drivers outlined in this report, which indicated that increases in adolescent pregnancy were due to religious beliefs, inadequate economic and social opportunities for youth, and desire to start a family. - We also provided evidence on the impact of COVID-19 on school drop-outs which have inequitably affected older female youth 17-19 years. - Together these additions help to provide further support for our claims that COVID-19 is anticipated to have a lasting impact on gender equality, SRH and female youth participation moving forward. |
| 15. In your limitations – you say that this research is rooted in heteronormative assumptions – and then define terms. Can you be explicit in how your research was rooted in these assumptions and why? Was anything done to counteract the impact of this? - I think it would be important to grapple with some of these ideas, considering the intention to “map the margins” in this research. Using Collins and Crenshaw, what are the limits of your ability to do this? Tying back to this would complete the narrative and show engagement with your work. | - We appreciate this comment, however, we focused largely on other dimensions of mapping the margins. We recognize that challenging heteronormative assumptions requires further attention not only in Malawi, but at global levels. Moreover, these issues were not discussed by any participants during focus groups or interviews. We highlight in the limitations that this is an area requiring future research and effort. - To briefly address this comment and show engagement with our postcolonial feminist theoretical framework we added: ***Adopting a postcolonial queer feminist perspective may be necessary to challenge the heteronormative structures which limit the participation of individuals who don’t fall within binary categories of sex/gender (96).*** - Reviewer #2 also suggested that we move the definition of heteronormativity to Table 1 (Key Concepts) and reduce our discussion of this section, these changes were made. |

| **Reviewer #2 Comment** | **Responses** |
| --- | --- |
| **General Comments** | |
| I have approached the article with an attitude of curiosity and good faith. I have offered extensive comments below because I believe in this author’s capability and the importance of the topic under examination. My main suggestion to the author is to draw out explicitly the creation and direction of power differentials in order to highlight how theory is played out in practice. As a medical anthropologist working in global health, I also want to see us collectively move beyond jargon in order to press the utility of these theories further. (This means owning one’s voice in new ways.) Doing so will move the article to another level. I note where this can be done in the appropriate lines below. The author has prompted me to think, and I want to return the favour. | - We are extremely grateful for the thoughtful and in-depth feedback provided by Reviewer #2. Further comments and responses are outlined, but we appreciate the curiosity and opportunity to strengthen our own critical thinking. |
| To move in this direction, I therefore ask for a gloss on several terms to point in that direction. For example, defining inequity (vs inequality) in the Table of Concepts would also help orient the reader to the author’s use of this often ambiguous term. I suggest placing this after ‘Gender’ and before ‘Gender Equity’ in the table. (I particularly liked the inclusion of a Table of Key Concepts.) | - Definitions of equity vs equality were added to Table 1. We will defer to the Editors whether this is repetitive with our definition of gender equality/equity. |
| In a more minor vein, the article would benefit from some tidying up. Style-wise, the author’s extensive use of “/” between words that are sometimes synonymous and sometimes rather distinct dilutes the author’s argument and reduces clarity for the reader. I’ve tried to suggest alternatives where appropriate. Though word count is not an issue for this journal, I have also tried to make those suggestions in ways that promote conciseness. | - Thank you for these suggestions, we have accepted those outlined. |
| Similarly, the author has used passive voice in several areas which diminishes the agency of the participants. I encourage active voice. In the press, use of passive voice is a tool of institutional oppression designed to exculpate institutional actors from any personalised, localized agency because it hides from view the actual subject creating or reinforcing oppressive structures. The passive voice can also deny visible agency to those of lower status for a similar reason: it obscures their own decision to act. Because of the author’s stated theoretical stance in decolonial feminist approaches to global health, I suggest the authors avoid use of the passive voice whenever possible. | - We greatly appreciate the thorough editing and suggestions and agree that passive voice undermines participants’ agency. We have made these edits throughout. |
| In the attachment, I listed my suggestions in line order, but have also indicated whether they concern ‘style’ (which includes any typos or grammatical oversights), ‘theory’, and ‘other’ in the lead of each suggestion. Theory and other are major points I believe the author should address before publication; style is minor, and simply a result of my close reading of the manuscript to improve clarity. | - Noted. In addition, comments from attachment are copied in the table below and responded in turn. |
| 7-9. *Style*: Consider breaking up or rephrasing the long sentence on lines 7-9.  20-22. *Style*: Rephrase to eliminate the ‘there is a lack’ and save words. Perhaps: “Moreover, to measure progress towards global objectives such as the SDGs, empirical evidence reflecting the everyday realities of youth is lacking, particularly in the global South.”  23-26. *Style*: This can be shortened to read simply: “Yet recent scholarship demonstrates that young women…”  29. *Style*: Eliminate the comma between ‘factors’ and ‘which’.  33. *Style and Theory*: ‘status’ suffices to encompass ‘power’ and ‘involvement’. Perhaps define either status or power in the Table of Concepts.  37. *Other*: Should the author cite (6) here, to advert the reader to previously published work derived from this study? Alternatively, cite that first paper in the Materials and Methods section.  54. *Style*: vulnerable to (not ‘vulnerable for’)  70. *Theory:* The listing of factors beginning in the 19^th^ century seems to follow a chronological format; yet the effects of (iii) Christianity seem to be given more space (and without complicating reception of its 19^th^ century Anglo-Protestant missionary forms, as contrasted with incipient feminism including within churches in Britain at this time, from what is inherent or enduring within Christianity as a two-thousand-year-old religion). Consider reorganizing the list to place the most salient factors first, or by placing line 77 – “Religion remains” as the first sentence after the list to signpost that the author will discuss that element specifically.  75. *Style and Theory*: I’m unclear on the author’s intended meaning. Does the author mean that Christianity crushed male-dominated practices? This does not match up with the sentence following. Revise for clarity, perhaps by drawing explicit attention to how factors (ii) and (iii) potentiated one another, reinforced one another, or had an otherwise synergistic effect. This, too, is an intersectional issue. The author has made a case for the importance of Christianity in terms of how it set up gendered divisions among those who create the structures which inform Malawian society today. Yet the author has also suggested that the history of gendered role divisions did not begin simply with the arrival of Christianity. I was left wondering first, what gendered divisions ‘traditionally’ existed in Malawi prior to the nineteenth century that the introduction of Christianity either promoted or sought to dismantle; and second, how much influence Islam (itself a fifteen-century-old religion) had on reinforcing or mitigating these same gendered practices. Was Islam introduced before Christianity? Did it orient society in particular direction when it was introduced? What about today? In terms of population, 15% seems a reasonably sized minority to influence social discourse – or is the community more closed in upon itself? Is legal pluralism recognized in Malawi?  98. *Theory*: The author mentions that some participants read this paper and offered feedback, which highlights their sense of self and collective action. Can PLOS give authorial credit to them, too?  103a. *Style*: Add ‘the’ to ‘inadequacies’.  103b. *Theory*: Perhaps ‘North American / Eurocentric’ or ‘white and western-centric’ is more exact. This is one case where the use of the “/” seems warranted. My experience is that many US feminists are not Eurocentric in the sense of being oriented towards Europe, but they are deeply informed by the authors’ experience of whiteness in North America and contextually by notions of US exceptionalism and power. (I was raised in Europe and came to North America in my late teens; the difference has always been apparent to me.)  105. *Style*: Eliminate ‘experienced by women’ since 1) the author has already established women as the topic earlier in the sentence, so this saves word count; and 2) the cited feminist writers also help influence and theorise the experience of men in post-colonial contexts, even if women are centred in the discourse. Women’s work doesn’t exist in a vacuum that never touches men. ☺  107. *Style and Theory*: ‘Systems of oppression’ has become mere jargon. Perhaps rephrase or elaborate with a gloss to highlight how intersectional theory conceptualizes the interlocking mechanisms by which some people and groups, like young people in this case, are denied power in decision making, opportunities, or whose influence over their own lives is otherwise marginalized. In other words, define the specific oppression which must be righted to be more just.  117. *Theory*: Perhaps add a citation to Geertz, *The Interpretation of* Cultures. He theorises culture as a system of constraints, and could be a useful starting reference if the author wants to focus on culture as a fluid system of oppression. (He was from the global North, even though writing from Indonesia and Morocco, so whether to citing him serves the team’s purposes is for the author to decide. Talal Asad has offered critiques of some of Geertz’s approaches, though not, to my knowledge around the idea of culture operating as a system of constraints. For additional critiques of Geertz, Ortner’s *The Fate of Culture* may prove useful.)  122. *Theory*: Perhaps cite Octavio Paz, *The Labyrinth of Solitude* for a Mexican comparison.  123. *Style*: “socio-cultural” will work.  123 – 127. *Theory*: YES! I would like to see more of this – perhaps even move this line (or a variation on it) up towards the introduction and abstract. (The author can augment citations here by referecing Bourdieu and Ortner’s critique of Bourdieu in *Anthropology and Social Theory* with respect to ‘growing into agency’.)  139. *Theory*: 1) Does this approach advocate for more than identifying subordination and instead also advocate for empowerment, recognition of agency, the ability to create space and shape one’s socio-political world? If so, state that because it seems to align with the author’s overall purpose in this article. 2) Does anyone also recognize the temporality of a ‘youth identity’? The idea that one grows out of ‘youth-hood’ simply because physiological time doesn’t cease and because of the coming-after-us of newer generations? Is this idea of temporal identity salient to the author’s point of illustrating how ‘youth’ is a very dynamic category?  140. *Theory*: After introducing intersectionality (line 103) and Murthi’s idea of difference as social position (line 132), perhaps press intersectionality towards the idea of position on a social field, defined by difference, which itself will vary by the context of the subjects who act on that field.  162: *Style*: Eliminate comma after ‘including’  162-164. *Other*: Will adding citations after some of these fields the author draws from be appropriate for the reader’s reference?  164. *Style*: Perhaps ‘intensive’, ‘time-limited’, or ‘temporally-circumscribed’ to eliminate the ‘/’?  165. *Style*: Eliminate the ‘and’ in order to begin a new sentence. This will maintain a quick rhythm for the reader.  166. *Style*: “our focus… illustrates” -- add ‘-s’ to ‘illustrate’.  174. *Style*: Eliminate ‘both’ from one of the two times it occurs.  181-188. *Style*: These three sentences could perhaps be combined by using the second sentence as the base and mostly eliminating the first and third sentences. This will buy the author word count. Currently, the three sentences seems repetitive for an audience of peers. The example which follows a combined sentence can do the heavy lifting by way of illustrating the meaning of a single sentence.  185. *Style*: Add a comma after ‘participants’.  189. *Style and Theory*: I would suggest ‘Canadian middle-class adult white female doctoral student’ to eliminate the commas, but perhaps the author’s point is to highlight each of these as discrete categories, rather than as producing an intersectional whole? Was JW raised middle-class? First generation college student? How stable is this identity? If it is dynamic, did that inform the choice of theory being deployed in this paper?  192- 194. *Style and Theory*: I think the author is trying to say they reflected on the decolonial imperatives facing researchers in the global north whose work is in the global south? Please clarify for the reader.  194. *Style and Theory*: Introduce the term ‘lived experience’ earlier in the paper (perhaps when the author mentions ‘lived context’), and gloss it with a single sentence. Cite Husserl, Merleau-Ponty, or another phenomenologist who coined the term, or a contemporary feminist scholar who took it up and deployed it in post-colonial contexts. Most of all, gloss it here or in the Table of Concepts – I can’t say how many times clinicians have asked me what it means.  195 - 196. *Style*: Eliminate ‘mixed’ and add ‘representative’ to ‘knowledge that extends’.  199. *Other:* How old are the community leaders? Are they youth leaders? Give me some quick background to position or contextualize these leaders in my mind.  202. *Style*: Eliminate comma after ‘Tumbuka’  203. *Style*: Eliminate ‘and’ to begin a new sentence. Saves word count.  210. *Theory and Other*: Are these six youth researchers mentioned among the authors?  213. *Theory*: Good job on capacity-building! What’s the follow-up to continue their growth in subsequent projects? In the body of the paper, the author has cited limits for this paper, but what’s next for those involved in the research? Doing this ‘leads by example’ – thank you for including it.  221-223. *Style*: This sentence repeats line 203. Consider eliminating or cutting down.  223 – 226. *Style*: Move up to follow line 199, for flow.  229: *Style*: The punctuation here is confusing. Revise for clarity. Perhaps: “Purposive sampling strategies employed snowball and ‘maximum variation’ sampling.”  233. *Other*: Open-ended ‘drawing’? Perhaps eliminate ‘open-ended drawing’ as ‘semi-structured interviews’ says plenty. Or, explicitly direct the reader to Table 3 for information about the visual method of gaining data via drawing.  233. *Style*: Add ‘either’ before ‘currently’ for clarity.  235. *Style*: For flow, start a new sentence after policy-making: “This latter group includes…”  236. *Style*: Eliminate ‘older’ and simply state the average for each group, then begin again with ‘most participants had some secondary education’.  240. *Style*: If all emails were via social media, save words by eliminating ‘email’.  242. *Style*: Eliminate the stray apostrophe on ‘participants’.  243. *Style*: Begin a new paragraph here.  245. *Style*: Cut ‘to gain diverse insights… discourses’ because the same information appears in line 247, about comparative contexts. Move that sentence, ‘Key informants provided’ up to follow ‘donors’. Alternately: merge the two sentences into a single sentence.  249. *Style*: Eliminate ‘and’ to begin a new sentence with ‘Thirty-two SSIs’.  Table 2. *Style*: Clean up the table a little bit. I suggest:   1) Mean Age: both sexes; male; female; range. 2) “Gender, *N*”, and male and female, not boys and girls since ‘garçon’ (‘boy’) was used for adult African men in their early twenties by French imperialists; it would also make the language consistent with the rest of table. 3) The ordering of rows between the two columns in the ‘District’ row does not match. Present the names of the communities in the same order (see Tufte, *The Visual Display of Quantitative Data*). Also, I would suggest either list gender break-outs in both columns or don’t mention gender. 4) Educational level achieved: shorten the descriptor to ‘lowest’ vs ‘highest’ for visual clarity. 5) For marital status, use a footnote or * in lieu of the parenthetical note, and add the * below the table, again for visual clarity   Table 3. *Style*:   1) Perhaps change ‘moderate participant observation’ simply to ‘observation’. 2) Clarify first bullet point in FGDs by using multiple sentences. 3) Reorganise second bullet point for clarity. 4) SSIs: eliminate parenthetical notes and add ‘with’.   265. *Style*: Revise to, “The analytic strategies we employed included…” Perhaps also use “document / visual analyses”, if appropriate.  266. *Style*: Eliminate the sentence, “Together these informed…” as it appears already earlier in this section.  277. *Theory*: What new theory did the author generate? So far, the author has only cited other theories. If the author did not create a new theory, stop the sentence at ‘themes’; the next sentence takes up conceptualisations. If the author derived a new theory, explicitly state it in the discussion or conclusion section, in the abstract, and perhaps in the Table of Concepts.  279. *Style*: Perhaps “policy strategies”?  281. *Style and Theory*: I’m not familiar with this visual analysis methodology but am curious about it. Can the author add a sentence more to describe it, beyond the citation and quote?  285. *Style*: Smoother: ‘how youth articulate their own experience of…’.  292. *Theory*: ‘Taboo’ has very specific anthropological associations (Durkheim, Malinowski, Mead, Douglas). Is this the best word to use, even with quotes? Perhaps ‘socially censured’ or ‘socially censurable’ (or even ‘prohibited’) is better?  296. *Style and Theory*: Use active voice: “Participants acknowledged”. This has the benefit of further centring their voices and not obscuring their agency. Also, I would favour either ‘attitudes’ or ‘norms’ over ‘attitudes/ norms’, since a ‘/’ already appears in this sentence.  297. *Style and Theory*: ‘Within society’ – yet the author has mentioned two distinct societies: the transnational society of international treaty-making, and the national society (or societies) in which such treaties are deployed. I suggest the author specify the level or levels they want to highlight to punch up their argument. Instead of continuing with ‘and SRH decision-making’, perhaps ‘as placed into sharp relief by SRH decision-making processes’.  298. *Style and Theory*: Perhaps continue the established international / national combination in lieu of global/ national? Otherwise, I would suggest ‘global / local’ as a more distinct category to use here.  299. *Style*: Add ‘the’ before underlying.  304. *Style and Theory*: Clarify what is meant by ‘gender mainstreaming’? Also, smoother: “in policies through public actions designed to achieve…”  305. *Theory*: “Polis” has a very specific connotation in political science. Does the author mean city-state? The political sphere? The ‘public forum’ in general? The polity? (cf. Hannah Arendt, *The Human Condition* for an accessible political theorist on this topic.)  306. *Style and Theory*: Use active voice to center accountability: ‘A public campaign for gender equity paradoxically illustrated inequity by advocating for 40/60 representation, with women lacking significant leadership positions within that proportion’. Passive voice is a tool of oppression designed to exculpate institutional actors from any personalised, localized agency. Therefore, avoid it whenever possible. ☺  316-318. *Style and Theory*: Experiencing gendered participation doesn’t clearly indicate they experienced gender disparities in access to both participation and leadership. Clarify.  322 - 325. *Theory*: The fact that reproductive health is a ‘women’s issue’ is highlighted by this. I suggest the author problematize that, perhaps by changing ‘gender imbalance / inequity’ to something like ‘the effects of this gendered assignment of health domains’? (Reproduction is a whole society, male and female, issue; gendering it as exclusively female does a disservice to the population as a whole. Additionally, leadership should not think that women leading in reproductive health suffices to give women a representative seat at the table, especially since it seems to further excuse men from taking responsibility for family health planning.)  338. *Style and Theory*: I suggest the author choose either oppressive or sexist. I think of sexist language as oppressive by its nature. Can the author provide a quote to highlight this? It will make the introduction of gerontocratic processes less sharp.  339. *Style*: ‘Gerontocratic’ comes out of nowhere. Although included in the Table of Concepts, introduce earlier in this paragraph as a gloss, or move this sentence and quote up to line 321 or 328.  346. *Style and Theory*:: Comma after ‘money’. I might also suggest digging a bit deeper to simplify this sentence: it is the donors’ money that gives them the power to impose the priorities of their political ideologies on local actors. *The flow of power is obscured* when each of these items is listed collectively. Highlight the directionality of the power differentials. The author has already mentioned capitalism in the introduction as disrupting previous stability in Malawi. Here’s the opportunity to make that connection clear again, and even compicate or expand a little on it.  347. *Style*: ‘programs’ – the already established topic of the sentence is policy-making.  348. *Style*: Perhaps ‘participation in SRH’ is smoother? Also, since clubs are organisations, go with orgs alone; remove the “/”.  346-348. *Style*: Move “Donor support” to follow the colon in line 345, then comment with the sentence beginning “their priorities”.  350. *Style and Theory*: Does the author mean, “the donor-driven nature creates a power asymmetry in SRH decision-making which inadequately reflects the local context”? The author’s theoretical sources would seem to point to making the creation and directionality of power imbalances obvious. Be explicit, since readers often skim work (even if reviewers do close reads).  358. *Style*: Development / implementation = introduction?  359. *Style*: ‘Submission of’. Also, change verb to active voice.  362. *Style and Theory*: Endorsed. ‘Not fully owned’ would imply the Foucauldian or Gramscian theory of résistance (which might make a good future paper for the author to consider: how local actors resist donor agendas). For additional citations, including Comaroff and Ortner, see <https://politicalanthro.wordpress.com/resistance/>  364. *Style and Theory*: Religious and traditional – otherwise, this sounds like leaders of traditional religions rather than the Christianity (and Islam) the author identified earlier as fostering patriarchy, allied with traditional ‘secular’ leaders in smaller communities (‘chiefs’).  371. *Style*: Remove ‘there remains’.  377. *Other*: It may be add to the impact of the quote to know whether this informant was male or female.  380. *Style*: Remove ‘the’ from ‘the opportunities’  386. *Style and Theory*: This informant notes cultural diversity in Malawi. Perhaps add a clause in the introduction to inform readers unfamiliar with Malawi’s mix of cultures about this social fact.  388. *Style and Theory*: Roles and status are distinct. Separate them with an ‘and’. (It means using ‘and’ multiple times in the sentence, so perhaps break the sentence up a bit: “shape the status of women from girlhood through adulthood, and the roles available to them in both family and community at each stage of life.”)  390. *Style*: Tighten: “women’s traditionally prescribed roles are outlined”  407. *Style*: Replace comma with semicolon before ‘however’. And YES – I was waiting for the keyword ‘naturalising’ (though it may need a gloss for readers unfamiliar with the term).  414. *Style*: ‘the confidence’; perhaps eliminate ‘peers and decision-makers’ as the quote is quite broad, as the author notes following it.  420. *Other*: Did anyone mention that men are not shy about discussing these things? If not, then it’s a double-standard – highlight.  422. *Style*: ‘or encouraged to censor’ (otherwise the sentence reads ‘discouraged from censor’.  424 - 433. *Other*: I think this is the key point of the section. Consider moving it up to make it the lead.  428. *Style*: discriminating against.  429. *Style*: Revise sentence for clarity. Restrictions required permission to participate?  432 – 433. *Style*: Tighten the sentence.  437. *Other*: Translation check: spoken > talked; otherwise, ‘talked about’  440. *Style*: ‘pre-pubescent girls’ suffices, as “girl” in this context implies “child”.  450 – 453. *Other*: I want to know more about this. Write another paper on this topic. ☺  457. *Style*: Add ‘that’ young women face, for clarity – or rephrase to ‘the SRH concerns young women face’  458. *Style*: comma before ‘and’.  462. *Theory*: Is the issue stigmatized or is the concern stigmatized? (This is a theoretical question: if the concern is stigmatized, that creates a different, more personal challenge in addition to the institutional barriers the author has cited.)  465. *Style*: Add an em-dash to indicate a break and restart of the speaker’s idea in this quote.  481. *Theory*: Is Malawi an honour-shame society, rather than a guilt-innocence society? If so, note in the introduction. The conflict between donors who come from guilt-innocence societies and local honour-shame societies is an overlooked conflict in the literature. (See Benedict, *Chrysanthemum and Sword* for more on honour-shame societies.)  533. *Style and Theory*: Use active voice, especially here where the topic is progress: the people most affected report progress.  544. *Style*: Replace / with ‘and’. If the author is concerned about word count: “Despite having vital roles in their families and communities…” and replace following despite with a synonym (‘even so’, ‘yet’ etc)  567. *Style*: ‘the 2017 amendments that raised…’  568. *Style*: ‘reveals a disconnect’ (remove ‘there is’ – ‘there is/ are’ is almost always a filler phrase which takes up precious word count space.)  569. *Style*: remove ‘and girls’’ since the team’s informants were all young women.  584. *Style*: Add comma after Canada.  585. *Style*: Replace ‘include’ with ‘such as’.  591. *Style*: Which? National, local, or both national and local?  594. *Style*: Add comma after active.  595. *Style*: Add ‘of’: the recognition and overcoming of gaps  596. *Style and Theory*: societal gender norms? Or social norms around gender? (The difference is a fine one.)  597. *Theory*: ‘Authentic’ is an existentially loaded term (cf. Kierkegaard, Sartre, Heidegger, Nietzsche). Unpack what authentic engagement concretely means to punch up the argument: that young people’s voices, and young women’s voices in particular, be centered in debates and discussed for their merits in light of the benefits accruing to society following their implementation, being brought on board to design implementation, etc – the author has plenty above, bring it back to the reader’s attention here, but be specific. This is a policy recommendation opportunity, after all.  600. *Style and Theory*: Acknowledge their engagement, both formal and informal.  604. T *Style*: ighten this sentence. ‘African critiques around the role and influence of foreign development donors reflects…’  609. *Style*: ‘Simultaneously’ will buy word count.  610. *Other*: Recently proposed?  613 – 615. *Style*: This sentence repeats 608 – 610. Eliminate one of the two.  617. *Style and Theory*: Change to active voice to own one’s own authorial advocacy here – if the author is the voice obscured by the passive construction. Alternatively, the author could use ‘we’ to generate buy-in among other global health actors.  618. *Style and Theory*: “Fund the implementation of.” Clarify the clause overall: when former colonial powers continue to implement their policies in liberated countries, those policies are automatically suspect as being a form of neo-colonisation.  619. *Style*: Choose between contraceptive or SRH for a more pointed argument.  621. *Style*: Politico-cultural realities  623. *Theory*: Is it worth pointing out that even when these foreign ideologies are promoted by women in the global north or west, it still erases the voices of women in the locales where these policies are implemented? Often, women in the west can take a gerontological view of women elsewhere – a ‘maternalistic’ (rather than paternalistic) approach that many in the global south find patronizing.  629. *Style*: Has  630 – 632. *Style*: The author’s argument is unclear here.  632 – 635. *Style*: This element seems better placed in the introduction section.  636. *Style and Theory*: Rather than by making structural change through political participation? If so, complete the thought by getting specific about the authorial team’s proposed alternative.  638. *Theory*: The author might also note the way capitalism creates a class incentive that pressures men to keep women out of competition, rather than fostering a more cooperative environment – after all, the male informant cited above essentially said, ‘young men and women need to be allies’.  647. *Style and Theory*: Use active voice – but I didn’t see the author cite any examples of this in the results section. Therefore, add to the results or cut from this section.  649. *Style*: ‘remains controversial’ suffices.  650. *Style and Theory*: “because [they will be considered – by whom? Because men and the older generation of men and women will call them] promiscuous or disrespectful of their elders for ‘voicing out.’” Changing the verb to active voice will strengthen the incisive quality of the argument and analysis.  651. *Style and Theory*: Use active voice: ‘The continued practice of traditional customs preserves patriarchy’ – but how traditional are these traditional practices? Earlier the author suggested it was 19^th^ century changes introduced by European powers which does this, meaning some of these practices are hardly ‘traditional’. (‘Traditional’ is therefore a contested term, explicitly own the authorial team’s position on what the term means, without needing to justify that position.)  652. *Style*: Switch ‘in addition to’ to ‘and their economic decision-making power’ to save on word count.  654 – 658. *Style and Theory*: Consider cutting this paragraph or focusing it strictly on ‘culture’, as the author didn’t really cite any informants discussing how religion posed barriers. I understand its purpose, but the author seems to unpack its point in the next section on patriarchy. Recall that patriarchy and gerontocracy are part of the culture under discussion, and have been since before the historical event of the colonial powers (history doesn’t begin with the arrival of Europeans). It may be that the informants are simply using the word ‘culture’ for ‘patriarchy’ and not separating out the two. A basic anthropological insight is that some societies split into two those notions that other societies see as intrinsically unified. In any event, if keeping this paragraph, split the sentence: “… insufficient. Challenging ‘essentialist interpretations’…”  660. *Style*: “Impact the opportunity and quality of”  663. *Style and Theory*: The author doesn’t go on to discuss post-colonial contexts, only patriarchal ones. Therefore, consider removing this sentence. The author can place the clause about social reproduction after the next sentence, as it ties the discussion back to earlier points the author makes.  666. *Style*: Contraception is part of SRH services, is it not? If so, remove and keep only SRH services.  666-668. *Style*: Consider moving this sentence later in the paragraph.  670. *Style and Theory*: The cited example contradicts tokenism, and evinces a sort of exasperation with it. The author could complicate the change dynamic by saying that this view doesn’t argue for women to participate on their own merits, on the simple fact that consideration of the total social fact means bringing women’s experiences to the table so that policies be informed by a fully representational judgement.  677. *Style*: Consider removing the first clause and beginning with ‘Older youth’ and ending with, ‘both when these situational factors are considered in isolation and more particularly when they intersect.’  677 – 688. *Style and Theory*: This part of the paragraph can benefit from a more tightly focused argument. I see the progression of the argument as: Age 🡪 Age + gender 🡪 complicated by temporality of these identities in the face of social expectations = social expectation of marginalization. However, I’m confused by the meaning of ‘ambiguity’ in line 686, and the use of ‘Despite this’ as a transition which seems on the contrary to support the preceding sentence. I see what the previous sentences are trying to build towards when I get to line 688, but it’s not there yet. The author has shown they have the skills already to take it there. Go for it!  694. *Style*: “Participants evinced consensus” (= remove ‘there is’) See Garner, *The Winning Brief*, or Guberman, *Point Made* for more on why ‘there + to be’ is superfluous in writing.  697. *Style and Theory*: This is where alluding to honour-shame societies earlier on becomes useful.  701. *Style*: Democratise = an infinitive verb, not a gerund.  704. *Style*: Turn the parenthetical note into its own sentence.  711. *Style*: Choose. Is it both? Say both, but they are not similar enough to be associated with a “/”.  712. *Style*: Consider eliminating the comma after passionate.  716. *Style and Theory*: I suggest the author choose experiences alone, since experiences produce identities in a recursive relationship. An additional reason to favour experiences is because the point of the sentence is the disruption of political systems which do not take a representative enough range of experiences into account, hobbling their political judgement. Identities are for politicking; experiences are for political decision-making. (This view of judging and politics is very Arendtian in perspective.)  726. *Style*: Strike ‘their’ and retain only ‘difference’.  731. *Style*: Strike constraints, since the norms which are to be changed are the constraints in question.  735. *Style*: Strike active, since the author has argued that ‘meaningful’ = active.  738. *Style*: For youth participation, not in. Clarify.  739. *Style*: Consider moving this sentence early on in the introduction, as it will orient the reader to the entire paper.  745. *Style*: ‘contraception access’ saves word count.  746. *Style*: Add Oxford comma.  750. *Style and Theory*: ‘Participation’ rather than ‘participatory rights’ may suffice, and save words. The author also has not earlier stated that the argument is rights-based as such; instead the argument seems more about ensuring equity, which can be done with or without relying on a rights-based discourse. See Moyn, *The Last Utopia*.  759. *Style*: Practises suffices.  760. *Style*: Remove ‘cultural’ as redundant in this sentence.  761. *Style and Theory*: Change to active voice. ‘We anticipated that the geographic range of sampling would capture cultural variation within the country.’  765. *Style and Theory*: As a gay man, I appreciate this paragraph. However, the author has already addressed it by saying they recruited few self-identified LGBTQI persons. I would suggest cutting this paragraph down to just lines 771 – 777, and placing ‘heteronormative’ into the Table of Concepts, earlier.  790. *Style and Theory*: Change to active voice. Who needs this, and for what purpose? Getting specific will make the argument more direct.  791. *Style*: Split the sentence after the citation. “…’borders’. The lessons…” | - Simplified sentence by removing “from community to international levels” - Done. - Change not made, as the first part of the sentence highlights the specific gap in evidence related to *young women’s* participation (previous sentence relates to scholarship on youth participation in general). - Done. - Removed power and involvement in this sentence. We also added our understanding of "power” to Table 1. - We have now cited our previously published paper as suggested. - Done. - Sentence on line 77 was moved as suggested. - This sentence was clarified. We have also added a sentence to outline women’s traditional roles prior to the 19^th^ century: ***Traditional Chewa women were afforded higher status as the “root of a lineage, the woman was seen as a sacred vessel of life” as their power and personhood was intimately related to their ability to bear children (35).*** - We added some details on the introduction of Islam to Malawi (page 8), however, emphasize that the European/colonial introduction of Christianity is largely implicated in the erosion of women’s status. Further exploration of the impact of Islam on social policy is beyond the scope of this paper. - All three Malawian co-authors (including one youth researcher) read and provided critical feedback on the manuscript. - Done. - Thank you, this suggestion was adopted. - Removed. - Agreed, in this instance we aim to describe the ways that intersectionality theory is taken up by postcolonial scholarship and rationalize our use of PCF vs intersectionality theory in this study. As the reviewer highlights (and commends) below, we have in turn outlined the ways we have understood and applied this theory in our own work. To address this feedback we added examples of the oppression experienced by youth (page 9) - …intersectionality conceptualizes the interlocking systems of oppression ***experienced by youth due to their social location (e.g., age, gender and class)…*** - Although we of course recognize Clifford Geertz’s crucial work , our understanding of “culture” in this piece is largely grounded in the work of post-colonial feminist theorists, and builds upon the insights, especially, of Jamaican-born (later British) Stuart Hall. In this regard, we appreciate the reviewer’s suggestion of referencing one of the key thinkers situating our theoretical stance and PCF approach. - Thank you for the interesting suggestion, which we will contemplate for subsequent works. - Done - We greatly appreciate this suggestion, however, Bourdieu and Ortner are not particularly well-aligned with other authors and works cited from our postcolonial feminist perspective. - We have added the following sentence to address this comment: ***It recognizes young people’s agency, expressed as collective action and participation against oppression, to create space and shape their socio-political worlds (18).*** - Few participants spoke of the temporality of “youth” as an identity, however, several scholars have discussed that “youth” often represents a dynamic and socially constructed category, shaped by diverse political, social, historical, and economic contexts. We added the following sentence and cited research from scholarship on pluralism of childhood in sub-Saharan African on page 10 to highlight this tension: ***Moreover, we understand the concept of “youth” as a dynamic and evolving category, shaped by the plurality of historical, political and social contexts and experiences (52).*** - In this discussion of “differences” we are outlining that young people’s differences represent assets to their citizenship (as per the difference-centred citizenship theory by Moosa-Mitha). We have added that these differences vary by context on page 10. - Done - This sentence was removed as the literature is outlined in the introduction, background and discussion more extensively. - Done - Done - Done - Done - We have combined the two first sentences to now read: ***Reflexivity about our “creative presence” (55) as researchers conducting a critical ethnography in Malawi underscores the importance of exploring the “complex (and sometimes contradictory) perspectives on privilege and difference” (54).*** - Done - Suggestion adopted. - We clarified and this now reads: We also grappled with our positionality and ***the decolonial imperatives facing researchers from the global North working in in the global South***, as we aimed to conduct ethical and deeply reflexive research, which promotes and (re)presents young people’s lived experiences of SRH participation. Therefore, we aimed to conduct ethical and deeply reflexive research which promotes and (re)presents young people’s lived experiences of SRH participation. - A brief definition of “lived experience” was added as suggested on paged 13-14. We cited a qualitative methods text, rather than phenomenologists. The following was added: ***Lived experiences relate to young people’s unique representation and understanding of experiences, choices and options, and their perception of knowledge; it is shaped by diverse factors including race, class, gender, sexuality, religion, and other elements which impact their everyday realities*** (61). - Done. - These were adult, community and traditional leaders. This was clarified on line 283, and now reads: … interviews with ***adult community and traditional leaders.*** - We also provided examples to describe the community and traditional leaders later on page 16 (***e.g., religious leaders, traditional chiefs or traditional/sub-traditional authority leaders)*** - Done. - Removed the rest of this sentence as it was repetitive (as per Reviewer #1 comment). - One of the youth researchers is a co-author, all youth researchers are recognized in the acknowledgements. - Since completing this project, the first author (JW) has served as a reference to several youth researchers and provided guidance/support for their involvement in various research project grants and/or scholarship applications. Most youth researchers have pursued other employment/educational opportunities. Although this discussion is beyond the scope of this paper, we plan to write a future reflexive account of our power and positionality in conducting this work. - Done - Done - Done - Open-ended drawing represents a separate, complimentary research method which we feel is necessary to also include here. However, to improve clarity we have expanded and indicated “semi-structured interviews with *an* open-ended *exercise*”. - Done - Done - Done - Recruitment was done by both email and social media (e.g., WhatsApp). This was clarified. - Done - Done - Done - Done - These suggestions were adopted. - 1) Moderate participant observation is a specific kind of participant observation that was conducted (reference is linked to authors that define/describe this type of work). No change was made - 2) Done - 3) Done - 4) Done - Adopted suggested phrasing, but did not put document/visual analyses in quotes, as these were standalone methods employed. - Removed from analysis section and moved to introduce the data generation methods. - We have removed “conceptualizations and theories” in this sentence to clarify. This largely referred to the “theorization” of our data - to view our data in “more abstract terms related to difference (rather than to their immediate substantive character)” (Eakin & Gladstone, 2020, p.2). - This change was not made, as some documents analyzed were policies (e.g., National Youth Policy) and some were “strategies” (e.g., the Youth-Friendly Health Services Strategy 2015-2020 and the National SRHR Strategy 2017-2022). To clarify the “/” was removed and now reads “policies and strategies”. - We added a sentence to describe the overall approach of the methodology. We added: We also employed a critical visual methodology to ***analyze drawings and reflect upon the participant, researcher and drawing.*** - Done   We agree, and have added a discussion on our use of these terms in the reflexibity section on page 15. We have added: ***We also critically reflect on the language employed throughout our work. For example, the terms “stigma” and “taboo” represent popular, often naturalized terms in SRH literature. Despite substantial variation in how “stigma” is defined and applied to substantive areas of inquiry, it is also critiqued for its individualistic focus, limited consideration of structural influences and connection of social labels to “undesirable” attributes to separate “us” from “them” (Link & Phelan, 2001, p. 370). The use of “taboo” is also inherently problematic, often defined by society and social norms to describe and control the “permitted and the prohibited, the do’s and don’ts” (Farberow, 1963, 1-2 as cited in Evans et al., 1999, p. 295). Although, in this study, both youth and key informant participants frequently employed these terms, we have chosen to employ these terms using quotations to reflect these challenges and to critically interrogate their meaning.***   - Both of these changes were made - This sentence was clarified to highlight the “global” rhetoric and more national commitments towards gender equality. - We have used “global/local” as suggested. - Done - We clarified our use of “mainstreaming” to describe the integration of gender in policies. We did not adopt the suggested change as it is in both policies *and/or* public actions (not necessary policy through action). - Thank you for this resource! Indeed, we are referencing a more political sphere or public governance in this instance. - Thank you: this suggestion was made. - We have clarified by indicating that women experienced gendered *and inequitable* participation and leadership at all levels of governance. - Done - We removed ‘sexist’, and provided an example including a quote (moved from the discussion section to here). - Gerontocracy was introduced earlier in the paragraph as suggested. - Comma added. - To clarify the directionality of power differentials, *“local”* policies was added here. - Done - In this instance, we are referring to both young women’s SRH *and* their participation as two separate priorities and areas of funding. - Clubs was removed - This comment was difficult to follow, but we combined several sentences here. - Yes, this is our intended meaning. The suggested edits were adopted. - Done - Done, and active voice was also employed. - Thank you for this feedback and resources – a great future paper indeed! - This sentence was removed as per feedback from Reviewer #1 - Done - Added that the quote is from a *female* youth key informant - Done - We added (in bold/italic) to reflect the cultural mix in Malawi (citing the 2018 National Housing Census): In Malawi, both patrilineal and matrilineal structures exist ***amongst the 12 major ethnic groups*** (33)… - Done - Done - Semicolon added, naturalizing was not defined. - Done. - Thank you for this comment, we have added ***“However, young men were often able to openly discuss issues of SRH without discrimination.”*** - Done – this was changed to “encouraged to censor their participation and perspectives…” - This section was moved closer to the start of this section. - Stylistically, we chose to keep our original wording here. - Clarified. - Tightened, added a semi-colon to break up long thought. - This interview was conducted in English by JW and was not translated. No changes made here. - Done - Thank you for this comment – we will try ☺ - Done - Done - Replaced “concerns” with “issues” as discussing these topics represent stigmatized topics which are not to be discussed openly (especially with male elders). - Done. - Although this represents an interesting area, in discussion with Malawian co-author (LB), this remains beyond the scope of this paper. The dichotomy of honour-shame society (and its impact on the donor relationships) was not considered applicable for this piece. - Done. - The first suggestion was adopted, and the / was replaced. - Done - Done, thank you for this suggestion. - Done - Done - Done - We mean both local and national, and have clarified this sentence. - Done - Done - We have clarified. - We have added a semicolon after “authentic engagement” and added the following to address the reviewer’s feedback: ***“whereby young people’s voices, and young women’s voices in particular, are given equal space and consideration within SRH decision-making processes.”*** - This sentence was revised, and this suggestion integrated. - Done - Done - Yes, this edit was made. - Done - Thank you, we have edited this sentence. It now reads “***We expose and acknowledge***…” - Sentence was clarified - We focused this argument on SRH policies, and removed contraception here. - Done - We have highlighted the inadequacies of white/Western feminist perspectives in the theoretical approach section. However, the impact of female-specific donors (and their maternalistic perspectives) were not identified or discussed by participants in this study. - Done - Clarified. - The sentence “***Girlhood” discourses in the global South have contested the use of “empowerment” strategies as the main solutions to what are deeply rooted, systemic inequities (12).*** was moved to the introduction. - Done - We added “young men’s hostility *and capitalist, class-based competition…*” to address this comment. - We have added this to the results section as per Reviewer #1 feedback, and provided a quote from a community leader to support our interpretation (page 28). We have also edited to use active voice. - Done. - Clarified that it is the older (often male) community members that consider young women promiscuous and disrespectful. Verb was also changed to active voice. - Changes made to use active voice. - We have continued to use the term “traditional” here. Although in the background we highlight that women’s status was undermined in the 19^th^ century, we also indicate (and cite Semu 2002 and Phiri 2007) to highlight that this status did not “spare” Chewa women from experiencing patriarchal customs including initiation ceremonies and early marriages. - Done - Sentence was divided as suggested. - Done - Replaced “postcolonial” with “historical, social and economic contexts” to address this helpful feedback. - Yes, removed. - Moved down 1 sentence, as suggested. - In response to earlier feedback from Reviewer #2, this quote was moved to the results to illustrate an example of oppressive language and meaning employed by male youth participants. No further change was made here. - Thank you for this suggestion. This stylistic edit was not adopted, the original statement although lengthy more clearly communicated our argument. However, we divided this long sentence into two. - We have clarified and added the reviewer’s suggestion of “temporality of these identities”. We also added the following to more clearly outline our argument in this paragraph: ***Young women are often relegated to “second-class” citizen status due to their systemic discrimination and failure to protect and realize their basic participatory and SRH rights (Nyamu-Musembi, 2007). These shortcomings highlight the importance of challenging normative perceptions of young women’s status roles in society, by generating contextualized insights of young people’s lived experiences of SRH participation…****.Efforts are needed…* - Done - See above. - We have used “democratize” as suggested. - Done - Done - Comma removed - We appreciate this thoughtful discussion and have used “experiences” rather than social identities here as suggested. - Done - Done - Done - Done - We feel this sentence reflects our interpretation of the data and various discussions with youth (and key informants), and more adequately indicates the implications of our study rather than introducing our topic/study. - Replaced availability with accessibility to clarify, however this edit was not made. - Done - This feedback was addressed, and we have used “participation”. - Done - Done - We used active voice, but kept some of the detail in our original sentence. - Heteronormativity was added to Table 1 (Key Concepts), removed sentence on heterosexuality. However, we kept the first sentence in this paragraph to help with flow and to orient the reader and added further detail to respond to Reviewer #1 feedback here. - This change was made, and sentence clarified. - Done |
